# Non-Iridescent Metal Nanomesh with Disordered Nanoapertures Fabricated by Phase Separation Lithography of Polymer Blend as Transparent Conductive Film

**DOI:** 10.3390/ma14040867

**Published:** 2021-02-11

**Authors:** Xinyu Chen, Yuting He, Xiaofeng Chen, Chunyu Huang, Yang Li, Yushuang Cui, Changsheng Yuan, Haixiong Ge

**Affiliations:** 1 Department of Materials Science and Engineering, College of Engineering and Applied Sciences, National Laboratory of Solid State Microstructures, Nanjing University, Nanjing 210093, China; cxy1296@163.com (X.C.); ythe@smail.nju.edu.cn (Y.H.); 18260091854@163.com (X.C.); huangchunyu@nju.edu.cn (C.H.); csyuan@nju.edu.cn (C.Y.); haixiong@nju.edu.cn (H.G.); 2 State Key Laboratory of Materials-Oriented Chemical Engineering, College of Materials Science and Engineering, Nanjing Tech University, Nanjing 210009, China; 3 National Laboratory of Solid State Microstructures, School of Physics, Nanjing University, Nanjing 210093, China; 4 Jiangsu Collaborative Innovation Center for Advanced Inorganic Function Composites, Nanjing Tech University, Nanjing 210009, China; 5 Jiangsu National Synergetic Innovation Center for Advanced Materials (SICAM), Nanjing Tech University, Nanjing 210009, China

**Keywords:** transparent conductive film, phase separation lithography, non-iridescence, metallic nanomesh, nanofabrication

## Abstract

Metallic nanomesh, one of the emerging transparent conductive film (TCF) materials with both high electrical conductivity and optical transmittance, shows great potential to replace indium tin oxide (ITO) in optoelectronic devices. However, lithography-fabricated metallic nanomeshes suffer from an iridescence problem caused by the optical diffraction of periodic nanostructures, which has negative effects on display performance. In this work, we propose a novel approach to fabricate large-scale metallic nanomesh as TCFs on flexible polyethylene terephthalate (PET) sheets by maskless phase separation lithography of polymer blends in a low-cost and facile process. Polystyrene (PS)/polyphenylsilsequioxane (PPSQ) polymer blend was chosen as resist material for phase separation lithography due to their different etching selectivity under O_2_ reactive ion etching (RIE). The PS constituent was selectively removed by O_2_ RIE and the remained PPSQ nanopillars with varying sizes in random distribution were used as masks for further pattern transfer and metal deposition process. Gold (Au) nanomeshes with adjustable nanostructures were achieved after the lift-off step. Au nanomesh exhibited good optoelectronic properties (R_S_ = 41 Ω/sq, T = 71.9%) and non-iridescence, without angle dependence owing to the aperiodic structures of disordered apertures. The results indicate that this Au nanomesh has high potential application in high-performance and broad-viewing-angle optoelectronic devices.

## 1. Introduction

TCFs have driven intense research efforts because they serve as essential components for numerous optoelectronic devices like solar cells [1,2,3], and various display-related applications including touch panels [4,5,6], organic light-emitting diodes (OLEDs) [7,8,9], and flexible displays [10,11], all of which are in much demand [12]. ITO, which has been developed for more than fifty years, still dominates the field today because of its high optoelectronic performance. However, ITO suffers from several drawbacks such as its scarcity of supply, high cost, brittleness, chemical instability and low conductivity [4,12]. These disadvantages certainly limit its applications in flexible and durable optoelectronic devices. It is highly desirable to develop alternatives with both high electrical conductivity and optical transmittance to traditional ITO. In addition, mechanical flexibility, durability, ease of processing, and large-scale fabrication feasibility are expected in the next generation of optoelectronic devices.

Recently, as the rapid developments in nanomaterials research, emerging conducting materials, such as conducting polymers [13,14], carbon nanostructures(carbon nanotubes and graphene) [15,16], as well as metallic nanostructures (metallic nanowires [17,18] and patterned metallic films [19,20]) have been widely explored as potential candidates for TCF. Nonetheless, conducting polymers, have not achieved widespread applications in TCF due to their poor environmental stability and low conductivity; how to make it in large scale and at low cost have remained challenges for carbon nanostructures; and metallic nanowires have inherent limitations of significant surface roughness and uncontrollable uniformity [12]. Compared with these materials, patterned metallic films offer simultaneously good electrical conductivity, optical transmittance, flexibility and durability [21,22]. Generally, patterned metallic films for TCFs consisting of periodic micro or nanostructures have been fabricated by means of conventional techniques e.g., nanosphere lithography [22,23,24], photolithography [25] and nanoimprint [26]. In addition to the complexity and high cost of the fabrication, the iridescent structural color could be observed when altering the viewing angle or the incident light angle, which has been attributed to the optical diffraction through constructive interference of scattering from periodic nano- and microstructures. The inherent characteristic could limit its utilizations in display-related optoelectronic devices that require broad viewing angles [27]. Low manufacturing cost and high transmittance without iridescent effect must be taken into account in the designing and fabrication of metal nanomesh. Random and disordered nanostructures can eliminate the iridescence phenomenon caused by the periodic nanostructures. Au nanomesh with disordered dual-size apertures was fabricated by packing dual-size nanoparticle mixture as a shadow mask for Au deposition. The Au nanomesh film showed excellent angle independent reflection properties as well as good optoelectronic performance [28].

In our recent work, we developed a new maskless lithographic approach, phase separation lithography based on polymer blends [29,30]. This approach is compatible with existing nanofabrication techniques to further transfer the phase separated nanostructures to aimed substrates. Compared with traditional fabrication methods, the phase separation lithography is an extremely simple, low-cost, and easily accessible methods for scalable fabrication of randomly but uniformly distributed nanostructures with average domain size down to 100nm. In this paper, we demonstrate a transparent, conductive, flexible and durable metallic nanomesh film with amorphous nanoapertures made by phase separation of PS/ PPSQ polymer blend. The formation of PS/PPSQ phase-separation on flexible PET substrate only involves a single spin-coating step. After phase separation, the PS constituent is selectively removed by O_2_ RIE because of the high etching resistance of the silicon-containing moiety of PPSQ. The remained PPSQ nanopillars with varying sizes in random distribution can be used as masks for further etching and deposition process. The metallic nanomesh film with amorphous nanoapertures is finally formed after removal of the depositing metal bulk. The sheet resistance and optical transmittance are optimizable by adjusting experimental parameters and the as-fabricated Au nanomesh TCF presents good optoelectronic properties (R_S_ = 41 Ω/sq, T = 71.9%) as well as benefits of amorphous nanoapertures eliminating iridescent effect.

## 2. Materials and Methods

### 2.1. Materials

All materials are commercially available and were used as received without further purification. PS (Mw = 100,000, Mw/Mn = 1.06) and PMMA (Mw = 996,000, Mw/Mn = 1.06) were purchased from Sigma-Aldrich (Shanghai, China). PPSQ was obtained from Gelest, Inc. (Morrisville, PA, USA).

### 2.2. Preparation of PPSQ/PS Blend Films

Polymer blend solutions with various concentrations and weight ratios were prepared by dissolving mixture of PS and PPSQ in toluene (concentration expressed as solute quality fraction at sum). The blend film was formed by spin-coating the solutions onto a prepared substrate at the speed of 3000 rpm.

### 2.3. Preparation of Metallic nanomesh films

PMMA solution was first spin-coated on a flexible substrate of PET as a sacrificial layer for lift-off. A 20 nm SiO_2_ layer was then deposited by plasma enhanced chemical vapor deposition (P80, OXFORD, Shanghai, China) at room temperature for 1 min. The phase separation procedure started with the spin-coating of PS/PPSQ polymer blend solution on the prepared surface. Afterwards, based on the high O_2_ plasma etching resistance of silicon containing PPSQ, the PS phase was selectively removed by means of O_2_ RIE with flow rate of 10 sccm, process pressure of 2 Pa, and radio frequency (RF) power of 30 W. Thereafter, A CHF_3_/O_2_ RIE (20/20 sccm, 2 Pa pressure, 40 W power) was used to eliminate the residual layer and the smaller domains that originated from PPSQ left in PS phase. A CHF_3_/CF_4_ RIE process (30/10 sccm, 2 Pa pressure, 40 W power) was used to etch the SiO_2_ layer. The pattern of PPSQ nanopillars of varying sizes in random distribution was then transferred step by step onto the flexible substrate after an O_2_ RIE (10 sccm, 2 Pa pressure, 40 W power) to remove the PMMA layer. Vacuum e-beam evaporation (ModelZZS500-2/D, Rankuum Machinery Ltd., Chengdu, China) was used to deposit Au and Ag film at a deposition speed of 1.0 Å/s. The thickness of the deposited film is controlled in the range of 5–40 nm. After metal deposition, the separated nanopillars were removed by a lift-off process of sonication in acetone for 3 min to form metallic nanomesh films.

### 2.4. Characterization

The morphologies of samples were characterized by using a field-emission scanning electron microscope (FE-SEM) instrument (ULTRA-55, Zeiss, Germany). Diameter and distribution of nanopillars were achieved via the software Nano Measurer V1.2.5 using the SEM image. Microscopic images were captured by a Microscope (Zeiss Imager. M1m, Zeiss, Germany). Sheet resistance of metallic nanomesh samples was measured by on a four-point probe (Summit1 1000M, Cascade, Fairview, OR, USA) recorded with a 2400 Sourcemeter (Keithley, Solon, OH, USA). The optical transmittance and total reflectance were detected on a UV−vis−NIR spectrophotometer (UV-3600, Shimadzu, Japan) in the wavelength ranging from 350 to 800 nm. The scattering and specular reflectance spectra were carried out through an angle-resolved microspectroscopy system (ARM, Fuxiang, China).

## 3. Results and Discussion

### 3.1. Strategy for Facile Nanofabrication of Metallic Nanomesh Film with Regulable Amorphous Nanoapertures

**Scheme 1:**
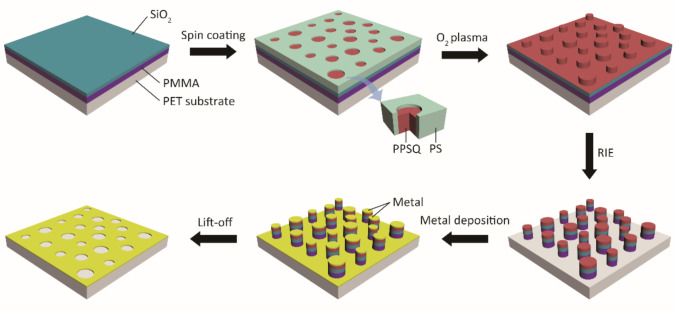
Schematic illustration on fabrication process of metallic nanomesh film with amorphous apertures.

Scheme 1 schematically shows the fabrication process of metallic nanomesh film with amorphous nanoapertures. In detail, PMMA solution was spin-coated on a flexible substrate of PET as a sacrificial layer for lift-off followed by the SiO_2_ deposition. Herein, the SiO_2_ layer played the role of preventing PMMA from dissolving in organic solution of PS/PPSQ polymer blend. The phase separation procedure started with the spin-coating of PS/PPSQ polymer blend solution on the prepared surface. Afterwards, the PS phase was selectively removed by means of O_2_ RIE based on the high O_2_ plasma etching resistance of silicon containing PPSQ. Thereafter, fluorine-based RIE was used to eliminate the residual layer and the smaller domains that originated from PPSQ left in PS phase, and the exposed SiO_2_ layer. The pattern of PPSQ nanopillars of varying sizes in random distribution was then transferred step by step onto the flexible substrate after an O_2_ RIE to remove the PMMA layer. A thin layer of metal was deposited onto the prepared structure by high-vacuum electron-beam evaporation. After a lift-off step, the metallic nanomesh film with amorphous nanoapertures was eventually formed. The largest size of samples fabricated in our experiment is 4-inch wafer which was determined by the limited size of spin coater and RIE chamber in the lab.

**Figure 1:**
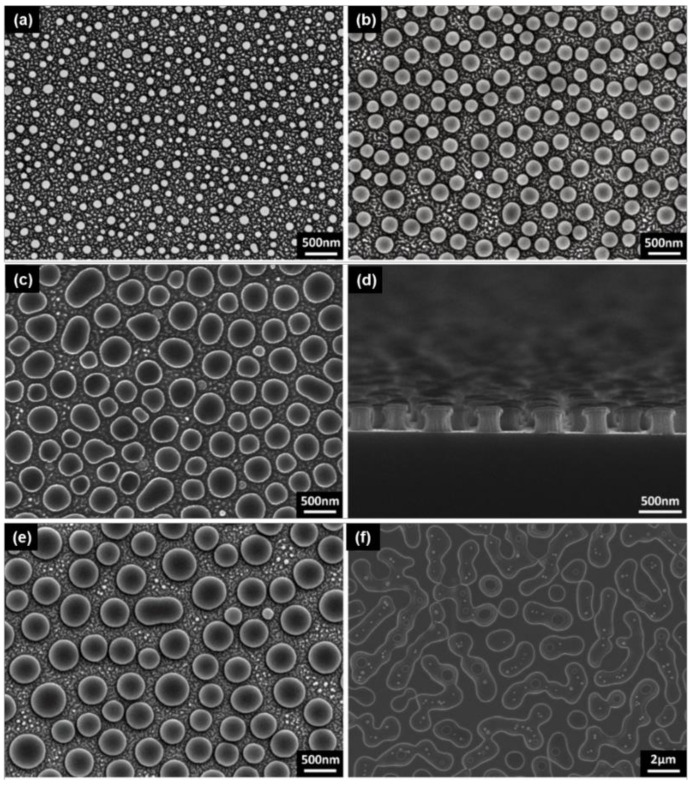
SEM images of different structures formed after phase separation and O_2_ RIE process with the PPSQ/PS ratio vary from (**a**) 1:4, (**b**) 2:3 to (**c**) 1:1. (**d**) cross-sectional SEM images the PPSQ nanopillars formed in (**c**) after removing the continuous PS phase and PPSQ remnants at 5 wt% concentration with PPSQ/PS ratio of 1:1. SEM images of different structures formed after phase separation and O_2_ RIE process with the PPSQ/PS ratio vary from (**e**) 3:2 to (**f**) 4:1 under the same experimental conditions of concentration (5%) and spin speed(3000 rpm). Note: the flocculent substance in micrographs is a few PPSQ remnants at the bottom.

In this method, PPSQ/PS polymer blends were selected as the materials for phase separation lithography due to their phase separation domain size in the range of 100s nm, which met the requirement of subwavelength apertures for metallic nanomesh. Moreover, the PS constituent without silicon element was selectively removed by O_2_ RIE and the PPSQ phase was remained for pattern transfer because of its high etching resistance of the silicon-containing polymers. The aspect ratio of the PPSQ phase can be further amplified by introduction of an organic transfer layer underneath the blend film, such as PMMA layer, through which low-aspect-ratio PPSQ patterns can be etched into high-aspect-ratio ones via a O_2_ selective etching process. The PMMA layer also took the role as a sacrificial layer for lift-off process to form the metallic nanomesh after metal deposition. The structural parameters of the metallic nanomesh, such as average pore diameter, metal wire width, duty cycle, were determined largely by the phase separation structures of the polymer blend. The pore diameter of the metallic nanomesh corresponded to the etched PPSQ domain size. Effects of the phase separation conditions, such as the weight ratio of PPSQ/PS, solution concentration of the polymer blend and the spin-coating speed, on the phase-separated morphology were investigated in our previous work [29]. The average size of the phase-separated domain can be tuned through an interplay of these factors. To simplify the experimental process, we fixed the spin speed at 3000 rpm and the concentration at 5% in this experiment. The geometry of the phase-separated nanostructures was mainly controlled by the variation of the PPSQ/PS weight ratio. Figure 1 shows the SEM images of phase separated PPSQ/PS blends after O_2_ RIE with different weight ratio of 1:4, 2:3, 1:1, 3:2 and 4:1 under the same experimental conditions of concentration (5 wt%) and spin speed (3000 rpm). When the relative concentration of PPSQ is small (ratio = 1:4, Figure 1a), we obtained small and sparse nanopillars. The selective removal of PS substantially enhanced the contrast of the phase separation morphologies for better SEM observation. With an increase of the PPSQ fraction, the domain size and area fraction of PPSQ obviously increased, as shown in Figure 1 and summarized in Table 1. For the blend with the smallest fraction of PPSQ (1:4), the SEM image (Figure 1a) shows that isolated circular nanopillars with an average diameter of 94 nm are sparsely distributed across the continuous surface. With an increase of the PPSQ fraction, the pillar size and area fraction of PPSQ obviously increased, as shown in Figure 1 and summarized in Table 1. The isolated circular nanopillars were transformed into a disordered bicontinuous morphology when the PPSQ fraction was further increased to 4:1. As the PPSQ/PS ratio close to 1:1, the phase-separated morphology exhibited an optimum PPSQ domain size and spacing between the PPSQ domains, which would correspond to the aperture size and wire width of the aimed metal nanomeshes. Figure 1d shows SEM image of cross-section of the nanopillars formed from the one with PPSQ/PS ratio of 1:1 after the phase-separated domains were etched into the PMMA layer. These nanopillars were used as a mask for subsequent metal deposition and lift-off process.

### 3.2. Influences of Thickness, Aperture Size and Duty Cycle of Metal Nanomeshes on Optical Transmittance and Sheet Resistance

**Figure 2:**
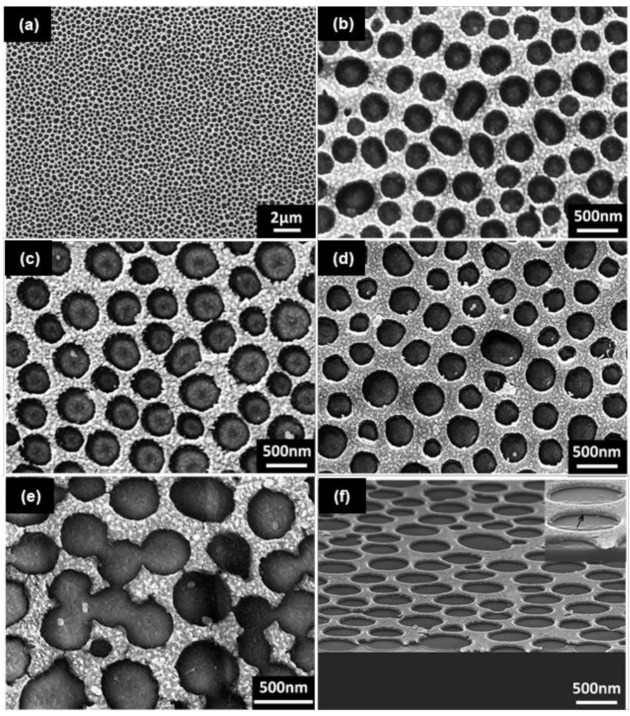
(**a**) SEM image of a large-area interconnected, smooth and uniform Au nanomesh film fabricated at 4wt% concentration at low magnifications. SEM images of our Au nanomesh films with amorphous apertures on a flexible PET substrate fabricated at the polymer blend solution concentrations of (**b**) 4 wt% and (**c**) 5 wt%. SEM images of Au nanomesh films fabricated at 5 wt% concentration with (**d**) redundant and (**e**) inadequate RIE time. (**f**) SEM image of Au nanomesh film on Si substrate at the tilt angle of ≈30° and the inset SEM image in (**f**) indicates the thickness of Au nanomesh film is about 30 nm. Note: Au nanomesh film thickness in (**e**) is 10 nm and others are 30 nm.

**Figure 3:**
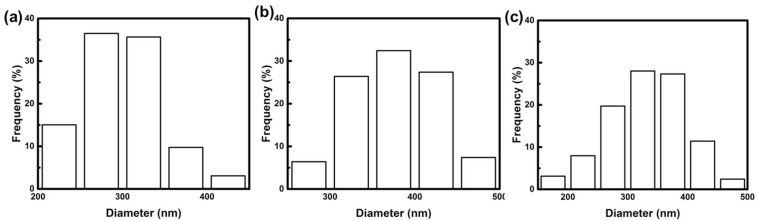
Diameter distribution of apertures in metallic nanomesh film samples with (**a**) polymer blend solution concentration of 4% and RIE time of 120 s, (**b**) polymer blend solution concentration of 5% and RIE time of 120 s and (**c**) polymer blend solution concentration of 5% and RIE time of 150 s.

Figure 2 shows SEM images of Au nanomeshes formed from the PPSQ/PS blend with ratio of 1:1 on a flexible PET substrate. As shown in the low-magnification top-view SEM image in Figure 2a, a continuous nanomesh film with disordered nanopores was successfully achieved. Since the diameter of the nanopillars (Figure 1d) can be gradually shrunk with the increase of etching time, the parameters of the nanomesh can be further modified via adjusting RIE time. Figure 2b–d are SEM images of Au nanomeshes formed with the nanopillars under different etching time, respectively. Table 2 and Figure 3 summarize the feature size distribution of apertures and average wire width of Au nanomesh film samples formed with various RIE time. The average aperture (pore) diameter of the nanomesh decreased from 376 to 300 nm, its average metal wire width increased from 90 to 160 nm, and its duty cycle (defined as the percentage of the area covered by the metal wires) increased from 43% to 61% as the O_2_ etching time increased from 150 s to 250 s. On the other hand, if the etching time was inadequate, some residual polymer layer would be remained between the isolated nanopillars, resulting in the merging of the isolated nanopores after lift-off process as shown in Figure 2e. Figure 2f shows Au nanomesh film with a thickness of approximately 30 nm on a silicon substrate instead of PET for better SEM observation of the cross-section of the nanomesh. The metal thickness of the nanomesh can be controlled by the metal deposition thickness.

**Figure 4:**
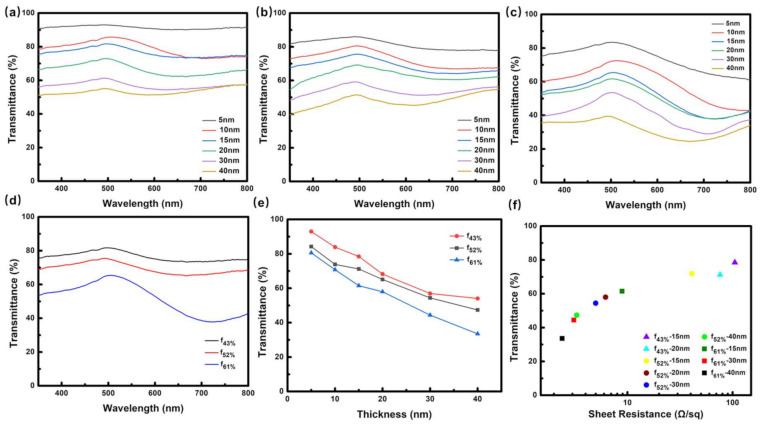
Optical transmittance (T) and sheet resistance (R_S_) of Au nanomesh films on the flexible PET substrate with different parameters, namely, duty cycle and metal film thickness. Optical transmittance spectra for the (**a**) f_43%_, (**b**) f_52%_ and (**c**) f_61%_ samples with various thicknesses. (**d**) Optical transmittance spectra of the 15 nm thickness samples with various duty cycles. (**e**) Optical transmittance comparison of Au nanomesh films with various metal film thicknesses and duty cycles. (wavelength at 550 nm) (**f**) Optical transmittance at 550 nm versus the corresponding sheet resistance for our Au nanomesh film samples (three pieces were made for each specific sample. The deviation of all samples were less than 1 Ω/sq).

**Figure 5:**
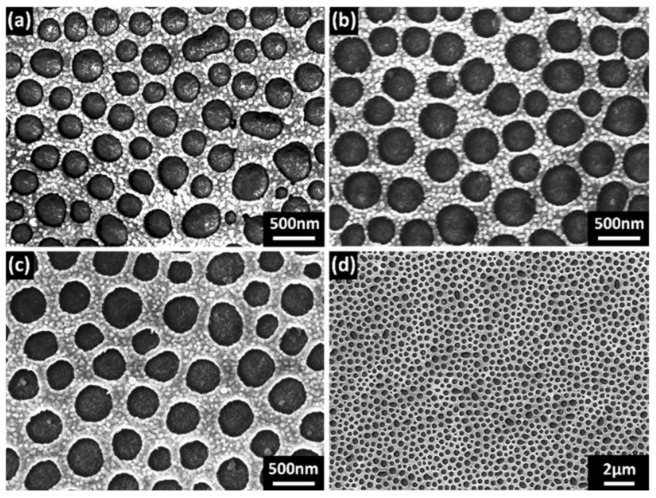
SEM images of our Ag nanomesh film samples (**a**) 1, (**b**) 2, and (**c**) 3. (**d**) SEM image of large-area Ag nanomesh film sample 3 at low magnifications.

Optical transmittance (T) and sheet resistance (R_S_) are two key and interrelated parameters for TCFs, and depend strongly on the metal thickness, the aperture size and duty cycle of nanomeshes. Generally, thicker metal thickness decreases the transmittance of the TCFs, while thinner metal thickness results in a poor electrical conductivity of TCFs. Figure 4 show the optical transmittance and sheet resistance of the prepared Au nanomesh on flexible PET substrates with different average aperture diameter, duty cycle and Au thickness. The samples were denoted as f_y_ (y = 43%, 52% and 61%, is the duty cycle of nanomeshes). Figure 4a–c are the optical transmittance spectra (in the range of wavelength from 350 to 800 nm) of the f_43%_, f_52%_ and f_61%_ samples with various thicknesses. As seen in Figure 4d–f, metal film thickness and duty cycle are two main factors to determine its optical and electrical properties. Along with increase of Au thickness or duty cycle, the optical transmittance is decreased whereas the lower sheet resistance is achieved. Therefore, the optical transmittance and electrical conductivity exhibit opposite trends for TCF materials. Figure 4f summarizes the experimental data, by plotting the optical transmittance at 550 nm versus the corresponding sheet resistance for our Au nanomesh TCFs. For the f_43%_ samples, with increasing Au thickness from 15 nm to 20 nm, optical transmittance decreases from 78.4% to 71.9% and the sheet resistance decreases from 105 Ω/sq to 41 Ω/sq. For f_52%_ samples, with increasing Au thickness from 15 nm to 40 nm, optical transmittance decreases from 71.2% to 47.4%, and the sheet resistance decreases from 76 Ω/sq to 3.3 Ω/sq. For f_61%_ samples, with increasing Au thickness from 15 nm to 40 nm, optical transmittance decreases from 61.5% to 33.5%, and the sheet resistance decreases from 8.9 Ω/sq to 2.4 Ω/sq. For the purpose of more promising uses in display-related applications, a low sheet resistance and a high optical transmittance are in demand for TCF materials. However, there is a tradeoff between these two contradictory parameters, that is the metal thickness are expected to be thin enough to transmit adequate light, while the thinner film causes poor electrical conductivity. For our case, when the metal thickness is thinner than 10 nm, the films looks cracked and discontinuous under the SEM observation as shown in Figure 2e, which makes the sheet resistance suddenly rise to approximately 10^3^ Ω/sq. By balancing metal film thickness and the duty cycle of samples, our Au nanomesh TCFs exhibit good optoelectronic performance (f_43%_ with thickness of 20 nm: R_S_ = 41 Ω/sq, T = 71.9%; f_52%_ with thickness of 40 nm: R_S_ = 3.2 Ω/sq, T = 48.4%; f_61%_ with thickness of 15 nm: R_S_ = 8.9 Ω/sq, T = 61.5%). Although optical transmittance of 48.4% is relatively low, it could be an excellent choice for high sensitivity touch panels with the compensation of backlight [4]. We also successfully fabricated Ag nanomesh film samples (Figure 5) with the sheet resistance lower to 1.2 Ω/sq, whereas their optical transmittance and durability are worse than that of Au.

### 3.3. Angle-Independent Non-Iridescent Metallic Nanomesh Films with Disordered Apertures

**Figure 6:**
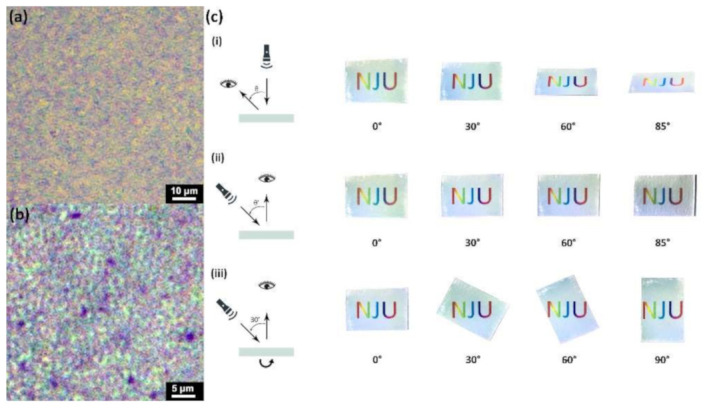
Microscopic images of Au nanomesh film (the f_43%_ sample) at (**a**) 500 and (**b**) 1000 times magnification. (**c**) Optical images showing the structural color of the Au nanomesh film when varying (**i**) the viewing angle, (**ii**) the angle of light incidence and (**iii**) the orientation of substrate (the incident light angle is fixed at 30°).

**Figure 7:**
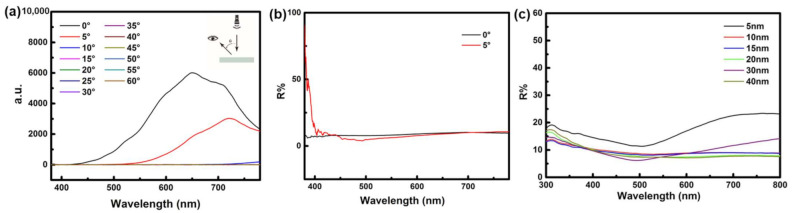
(**a**) Optical backscattering, (**b**) specular reflectance and (**c**) total reflectance spectra of the f_43%_ samples measured at different angles with fixed angle of incidence.

Different from traditional micro-patterned metal grids, which can be sensed by naked eyes, our metallic nanomesh TCFs has high clarity with nanoscale apertures and metal wires. Previously reported nanopatterned metallic films usually have apertures in periodic arrays, which could appear brilliant iridescent angle-dependent colors. This phenomenon is caused by optical diffraction. As the structural period of aperture arrays is comparable in size to incident light wavelength, the patterned metallic films can be regarded as a diffraction grating for visible light waves. The aperture arrays scatter light in all directions because of the refractive index differences between the apertures and the surrounding film, and then these scattered lights generate constructive interference and reach maximum intensity on account of the periodic structures, that depends on the specific viewing angle [28]. Instead, our metallic nanomesh films with disordered apertures can eliminate iridescent phenomenon caused by the optical diffraction of periodic nanostructures. The reason is that the random size and distribution of the disordered apertures can prevent constructive interference of the diffracted light for the formation of diffraction maxima. Optical images of Au nanomesh with disordered apertures are taken in various viewing angles, angles of light incidence and orientation angles of the nanomesh, and exhibit angle-independent reflection properties. As shown in Figure 6c, when the angles are altered in the range of 0–90°, respectively, there is no obvious change in the colors of the observed sample. The optical backscattering spectra measured at different angles with fixed angle of incidence further verified the angle-independent reflection properties. As seen in Figure 7a, the scattering intensities of measuring angles from 10° to 60° are too weak to read. The scattering intensities measured at 0° and 5° are high enough to read, the difference of peak position between which are small (from 650 nm to 720 nm). Due to the high transmittance of the sample (71.9%), as seen in Figure 7b, the specular reflectance spectra measured at 0° and 5° are also quite low (<10%). In addition, the total optical reflectivity spectra are all below 20% (Figure 7c). This indicates that the scattering of the disordered nanomesh is weak and angle-independent.

### 3.4. Flexibility, Durability and Application Examples

**Figure 8:**
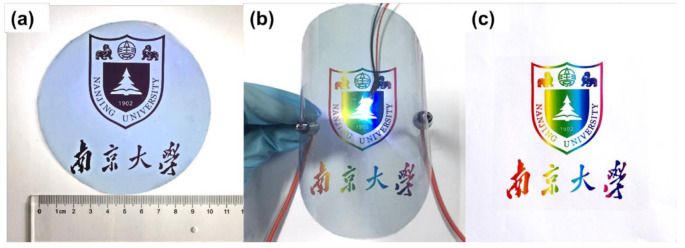
Photographs of the fabricated Au nanomesh film sample, indicating its (**a**) transparent and uniform over a large area and (**b**) excellent flexibility and electrical conductivity properties. (**c**) Photograph of the iridescent logo in (**b**) without the Au nanomesh film as a comparison.

**Figure 9:**
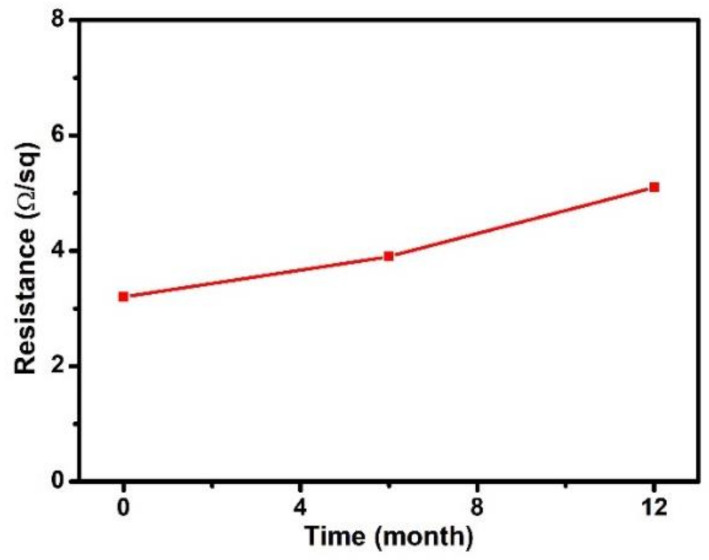
Sheet resistance at intervals of the Au nanomesh sample (R_S_ = 3.2 Ω/sq, T = 48.4%).

As shown in Figure 8, we successfully fabricated a wafer-scale, flexible Au nanomesh TCF. In our experiment, the maximum size of the Au nanomesh TCF is limited to 4. inch wafer by the stage size of RIE machine and spin coater. The sample kept transparent and uniform appearance over a large area (Figure 8a). Through it, an iridescent logo underneath can be seen clearly (Figure 8b,c). A LED bead was lighted up through the Au nanomesh TCF. Its brightness was not obviously attenuated even when the film is bent with a bending angle of 90°, indicating good flexibility of the prepared TCF (Figure 8b). In order to testify the durability of the Au nanomesh films, we measured the resistance of the sheet stored in an ambient environment with different storage time. The sheet resistance of these samples (Figure 9) remains nearly unchanged (from 3.2 Ω/sq to 5.1 Ω/sq) within the measurement error after a long time (12 months) storage.

## 4. Conclusions

In conclusion, we have developed a transparent, conductive, flexible and durable metallic nanomesh TCF with amorphous apertures by phase separation, which allows cost-efficient and large-scale fabrication on flexible substrates. The obtained metallic nanomesh contain random aperiodic structures, which can easily eliminate the iridescent color caused by the optical diffraction of periodic structures to possess an angle-independent structural color. Our Au nanomesh film presents good optoelectronic properties as well as high visibility, excellent mechanical flexibility and durability and can be widely used as a flexible and durable TCF in various high-performance and broad-viewing-angle optoelectronic devices.

## Figures and Tables

**Table 1:** Summarization of feature size distribution and area fraction of PPSQ nanopillar structures with various PPSQ/PS ratios and solution concentration of 5wt%. The number of particles and intervals counted is 150 for each sample.

PPSQ/PS Weight Ratio	Spin Speed (rpm)	Average Feature Size (nm)	Standard Deviation of Feature Size (nm)	Average Intervals (nm)	Standard Deviation of Average Intervals (nm)	Area Fraction (%)
1:4	3000	94	21	337	59	20
2:3	3000	226	41	133	65	39
1:1	3000	382	67	105	48	64
3:2	3000	412	61	138	67	58
4:1	3000	--	--	--	--	--

**Table 2:** Summarization of feature size distribution of apertures and metal wires in Au nanomesh film samples with various polymer blend solution concentrations and RIE time. The weight ratios of PS/PPSQ were all set as 1:1. The number of items counted is 100 for each sample.

Concentration (%).	RIE Time (s)	Average Aperture Diameter (nm)	Standard Deviation of Feature Size (nm)	Average Metal Wire Width (nm)	Standard Deviation of Average Intervals (nm)	Area Fraction (%)
4	120	300	46	90	55	52
5	150	376	58	112	54	43
5	250	333	65	160	57	61

## Data Availability

The data used to support the findings of this study are available from the corresponding author upon request.
